# Silencing of LncRNA C1RL-AS1 Suppresses the Malignant Phenotype in Gastric Cancer Cells via the AKT/β-Catenin/c-Myc Pathway

**DOI:** 10.3389/fonc.2020.01508

**Published:** 2020-09-02

**Authors:** Wu Zhen-Hua, Gong Yi-Wei, Zhao Li-Qin, Zhang Jie-Yun, Gong Zhe, Guo Wei-Jian

**Affiliations:** ^1^Department of Medical Oncology, Fudan University Shanghai Cancer Center, Shanghai, China; ^2^Department of Oncology, Shanghai Medical College, Fudan University, Shanghai, China

**Keywords:** lncRNA C1RL-AS1, malignancy, β-catenin, c-Myc, gastric cancer

## Abstract

**Purpose:** Numerous studies have shown that lncRNAs play vital roles in the development and progression of cancer. However, investigations of lncRNAs in gastric cancer are limited and need to be further pursued.

**Materials and Methods:** According to RNA-seq results of gastric cancer (GC) tissues, we identified a novel lncRNA, C1RL-AS1. qRT-PCR was used to detect the expression level of C1RL-AS1 in paired GC and normal tissues. Nuclear/cytoplasmic fractionation was applied to evaluate the distribution of C1RL-AS1 in GC cells. For functional evaluation, CCK-8, colony formation, transwell, and apoptosis assays were used to determine the oncogenic role of C1RL-AS1.

**Results:** C1RL-AS1 was upregulated in GC tissues, and high expression levels of C1RL-AS1 were associated with poor prognosis. Further *in vitro* functional assays revealed that silencing C1RL-AS1 attenuated the proliferation rate and migration ability and enhanced the apoptotic rate and the senescence of GC cells. The subsequent underlying mechanistic investigation revealed that Wnt/β-catenin was involved in C1RL-AS1-mediated signaling. Rescue experiments suggested that C1RL-AS1 probably promoted the malignant phenotype via the AKT/β-catenin pathway by downregulating c-Myc.

**Conclusions:** C1RL-AS1 probably exerts its biological function by mediating the AKT/β-catenin/c-Myc pathway, indicating a novel therapeutic target in GC.

## Introduction

Gastric cancer is one of the most common malignant tumors in humans; its incidence ranks fifth, and its mortality ranks third worldwide (1). In China, there were ~679,000 new cases and 498,000 deaths from gastric cancer in 2015 (2). Owing to the heterogeneity of the diseases, most patients are diagnosed at a late stage, and the 5-year survival rate is unsatisfactory. Thus, urgent efforts are needed to discover new biomarkers for the early diagnosis and treatment of gastric cancer (3).

Long non-coding RNAs (lncRNAs) are more than 200 bp in length with limited or no coding protein abilities (4, 5). Mounting evidence has revealed that lncRNAs participate in diverse intracellular activities, such as chromosome modification, genome modification, transcriptional activation, and intranuclear transport (4, 6). The aberrant expression of lncRNAs is usually associated with many biological events in cancer progression (7–9). For example, lncRNA ZNNT1 induces autophagy to inhibit tumorigenesis of uveal melanoma (10). LncRNA EPB41L4A-AS1 functions as a repressor of the Warburg effect and plays important roles in metabolism in cancer (11). Our previous study also showed that onclncRNA-626 promotes malignant behavior in gastric cancer by interacting with SRSF1 (12).

The Wnt/β-catenin pathway plays a significant role in the development and progression of cancer, especially in the regulation of metastasis (13). When the Wnt ligand activates the Frizzled and LRP receptors on the cell membrane, the APC/Axin/GSK-3β complex cannot phosphorylate and degrade β-catenin into the nucleus from the cytoplasm. The β-catenin in the nucleus can bind with TCF/LEF transcription factors and cause abnormal expression of downstream c-Myc or cyclin D1, which in turn promotes proliferation, metastasis, and other biological processes. Several studies have indicated the involvement of Wnt/β-catenin in the lncRNA-mediated pathway. For example, lncRNA DANCR promotes progression in cervical cancer via the Wnt/β-catenin pathway (14).

Here, we identified a novel lncRNA, C1RL-AS1, which probably exerts its biological function by mediating the Wnt/β-catenin pathway, indicating a potential therapeutic target for gastric cancer (GC) patients in the future.

## Materials and Methods

### GC Cell Lines and Reagent

The human gastric cancer cell lines SGC-7901, MKN-28, AGS, MGC-803, and HGC-27 and the normal gastric epithelial cell line GES-1 were purchased from the Shanghai Cell Bank Type Culture Collection Committee (CBTCCC, Shanghai, China). Cells were cultured in DMEM (HyClone, Logan, UT, USA) with 10% fetal bovine serum (Biological Industries, Israel) at 37°C under a humidified atmosphere with 5% CO_2_ as described previously. SC-79, an AKT activator, was purchased from Selleck Company (USA).

### Human GC Tissues

Ninety paired gastric cancer and normal tissues were obtained from Biobank in Fudan University Shanghai Cancer Center (FUSCC). All patients underwent gastrectomy from 2008 to 2010 in FUSCC and were diagnosed with gastric cancer. None of them received chemotherapy or radiation before surgery. The overall survival (OS) time was defined as the time from the date of surgery to the date of death, and disease-free survival (DFS) time was defined as the time from the date of surgery to the date of first recurrence. The clinicopathological features of the patients are shown in Table 1 according to the 7th version of the American Joint Committee on Cancer staging system. All procedures were approved by the ethics committee of FUSCC.

**Table 1:** Relationships between lncRNA C1RL-AS1 expression and clinicopathological features of GC patients.

**Parameter**	**No. of**	**C1RL-AS1**	**C1RL-AS1**	***P*-value**
	**patients**	**(low)**	**(high)**	
Age (years)				0.0566
<60	40	25	15	
≥60	50	20	30	
Gender				>0.9999
Male	69	34	35	
Female	21	11	10	
Tumor size				0.6732
≤5cm	47	25	22	
>5cm	43	20	23	
Histologic grade				0.8298
Moderately/Moderately-	36	19	17	
Poorly differentiated				
Poorly differentiated	54	26	28	
pT stage				**0.0433**
T1-3	30	20	10	
T4	60	25	35	
pN stage				**0.0352**
N0	26	18	8	
N1-3	64	27	37	
pTNM stage				0.1868
I+II	18	12	6	
III	72	33	39	

*The bold values indicate that the P values < 0.05*.

### Subcellular Fractionation

For nuclear/cytoplasmic fractionation, the PARIS kit was used, and 1 × 10^7^ HGC-27 cells were suspended in cell fractionation buffer according to the manufacturers' instructions. Then, RNA was extracted, and qRT-PCR was performed. GAPDH served as the cytoplasmic endogenous control, and U1 small nuclear RNA served as the nuclear endogenous control.

### RNA Isolation, Reverse Transcription PCR, and Quantitative Real-Time PCR

RNA isolation, reverse transcription, and qRT-PCR were performed as previously described. The relative expression of detected genes was calculated using the comparative CT (2^−ΔΔ^CT) method. The primers were as follows:

β-actin-F: GATCTTCGGCACCCAGCACAATGAAGATC,
β-actin-R: AAGTCATAGTCCGCCTAGAAGCAT;
lncRNA C1RL-AS1-F: GGCTCCCACTGATTCTACATTAGG,
lncRNA C1RL-AS1-R: TCCTTCTCCTTCTACTCACAGAGC;
18S-F: CGGACAGGATTGACAGATTGATAGC,
18S-R: TGCCAGAGTCTCGTTCGTTATCG;
GAPDH-F: AGAAGGCTGGGGCTCATTTG,
GAPDH-R: AGGGGCCATCCACAGTCTTC;
U1-F: GAAACTCGACTGCATAATTTGTGGTAG,
U1-R: CTTGGCGTACAGTCTGTTTTTGAAACTC.

### Plasmid, siRNA, and Transfection

c-Myc overexpression plasmid and the corresponding negative control plasmids were purchased from Vigene Biosciences (Shandong, China). The c-Myc plasmid was constructed in the pENTER vector. The information and cDNA sequence of pENTER is listed in Supplementary Materials and Methods II. The siRNA and ASOs (antisense oligonucleotides) of C1RL-AS1 were designed and synthesized by RiboBio (Guangzhou, China), and the sequences were as follows: si-C1RL-AS1, GCTGCTGTATTCGTCCATT; and ASO-C1RL-AS1, CACGCACCGTACATTGAAGA (Supplementary Materials and Methods I). Lipofectamine 2000 (Invitrogen) was applied to transfect the aforementioned plasmids and siRNAs into gastric cancer cells according to the manufacturer's instructions.

### Cellular Senescence

The procedures of cellular senescence were performed as previously described according to the manufacturers' instructions (15). In brief, 48 h after transfection with C1RL-AS1-ASO, cells were washed with phosphate-buffered saline (PBS) and fixed for 15 min. Then, the cells were incubated overnight at 37°C by staining with dye solution. Finally, the cells were counted under a microscope, and the senescent cells were indicated in blue.

### Proliferation and Colony Formation Assay

For the proliferation assay, 2,000 cells were seeded in a 96-well plate after the indicated treatment. On the next day, cell viability was detected by Cell Counting Kit-8 (CCK-8) assay (Dojindo, Japan) according to the manufacturer's instructions. Each experiment was conducted in triplicate, and cells were measured continuously for 5 days.

For colony formation, ~1,000 cells were seeded in a six-well plate with complete medium and grown for nearly 2 weeks. After that, visible colonies were fixed with 4% paraformaldehyde, stained with 1% crystal violet, and counted.

### Migration Assay

A total of 4 × 10^4^ transfected HGC-27 or AGS cells were suspended in 200 μl FBS-free DMEM medium and seeded into the upper chamber of a transwell insert (24-well insert; pore size, 8 μM; BD Biosciences), and 600 μl 10% FBS DMEM medium was added into the lower chamber. After incubation for 24 h, the migrated cells were fixed with 4% paraformaldehyde, stained with 1% crystal violet, and counted.

### Western Blot

The western blotting procedures were described previously (16). The primary antibodies used were as follows: E-cadherin (1:1,000; Cell Signaling Technology), Snail (1:1,000; Cell Signaling Technology), Slug (1:1,000; Cell Signaling Technology), AKT (1:1,000; Cell Signaling Technology), p-AKT (Ser473) (1:1,000; Cell Signaling Technology), p-GSK3β (Ser9) (1:1,000; Cell Signaling Technology), β-catenin (1:1,000; Cell Signaling Technology), c-Myc (1:1,000; Cell Signaling Technology), p16 (1:1,000; Cell Signaling Technology), p21 (1:1,000; Cell Signaling Technology), p53 (1:1,000; Cell Signaling Technology), GAPDH (1:2,000; Proteintech Group), PCNA (1:1,000; Proteintech Group), and β-actin (1:2,000; Cell Signaling Technology). The secondary antibodies were as follows: goat anti-rabbit and anti-mouse IgG (1:10,000 each; Jackson ImmunoResearch Laboratories).

### Cellular Apoptosis Analysis

The effect of C1RL-AS1 on apoptosis was detected by flow cytometry staining with Annexin PE/7AAD (BD Pharmingen). In brief, cells were cultured for 48 h after transfection with C1RL-AS1 ASO, harvested, and washed twice in PBS. After that, a total of 2 × 10^5^ cells were resuspended in 100 μl binding buffer, and 5 μl Annexin PE and 5 μl 7AAD were added. After 15 min of incubation in the dark, flow cytometry was performed.

### Statistical Analysis

Data are shown as the mean ± SEM from three independent experiments using GraphPad Prism 7.0. Student's *t* test was applied to compare the differences between different groups. Fisher's exact test was used to analyze the relationship between clinicopathological features and C1RL-AS1 expression. Pearson's correlation was employed to analyze the correlation between C1RL-AS1 and c-Myc expression in GC tissues. *P* < 0.05 was considered statistically significant.

## Results

### LncRNA C1RL-AS1 Was Upregulated and Correlated With Prognosis in GC

**Figure 1:**
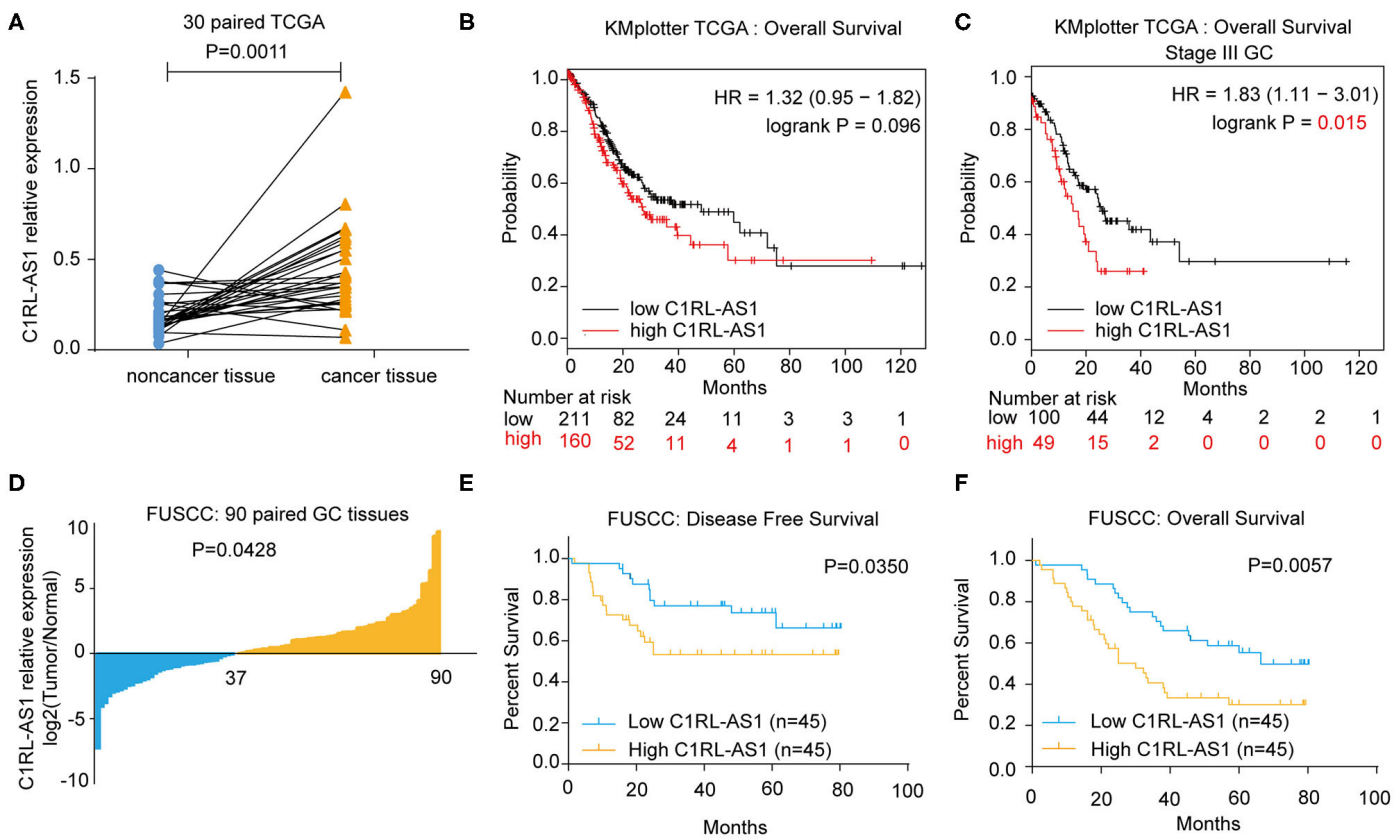
**(A)** LncRNA C1RL-AS1 expression was evaluated in 30 paired GC patients in the TCGA cohort. **(B)** Overall survival analysis according to C1RL-AS1 expression in GC patients in TCGA from Kaplan–Meier Plotter. **(C)** Overall survival analysis according to C1RL-AS1 expression in stage III GC patients in TCGA from Kaplan–Meier Plotter. **(D)** qPCR was applied to detect the fold changes in lncRNA C1RL-AS1 expression in 90 pairs of GC and adjacent normal tissues. **(E,F)** Analysis of the association between lncRNA C1RL-AS1 expression levels and disease-free survival (DFS) and overall survival (OS) time in 90 GC patients.

To uncover novel lncRNAs in GC, RNA-seq was employed in six paired GC (lymph node metastatic in pathology) and normal tissues. LncRNA C1RL-AS1 was dramatically upregulated according to the RNA-seq data (log_2_FC = 1.744, *p* < 0.0001). Next, C1RL-AS1 expression was evaluated in gastric cancer data from the TCGA database. As shown in Figure 1A, C1RL-AS1 was significantly upregulated in 30 paired patient samples in the TCGA (*p*=0.0011). Although a trend difference in the survival curves between the C1RL-AS1 low and high groups in Kaplan–Meier Plotter (17) was observed, the difference was not statistically significant (*p* = 0.096, Figure 1B). Intriguingly, a significant difference in survival curves was observed between stage III GC patients with high C1RL-AS1 levels and those with low C1RL-AS1 levels (*p* = 0.015, Figure 1C).

To further validate the clinical significance of lncRNA C1RL-AS1 in GC, qRT-PCR was performed to detect C1RL-AS1 expression in 90 paired GC and normal tissues from FUSCC. Consistently, the expression level of C1RL-AS1 was increased in gastric cancer tissue compared with adjacent tissue (Figure 1D, *p* = 0.0428). Subsequent survival analysis revealed that patients with high expression of C1RL-AS1 had shorter DFS time (*p* = 0.0350) (Figure 1E) and OS time (*p* = 0.0057) (Figure 1F) than those with low expression. In addition, the clinicopathological features analysis in Table 1 showed that C1RL-AS1 expression was correlated with pT stage (*p* = 0.0433) and pN status (*p* = 0.0352).

### The Characteristics of LncRNA C1RL-AS1 in GC

**Figure 2:**
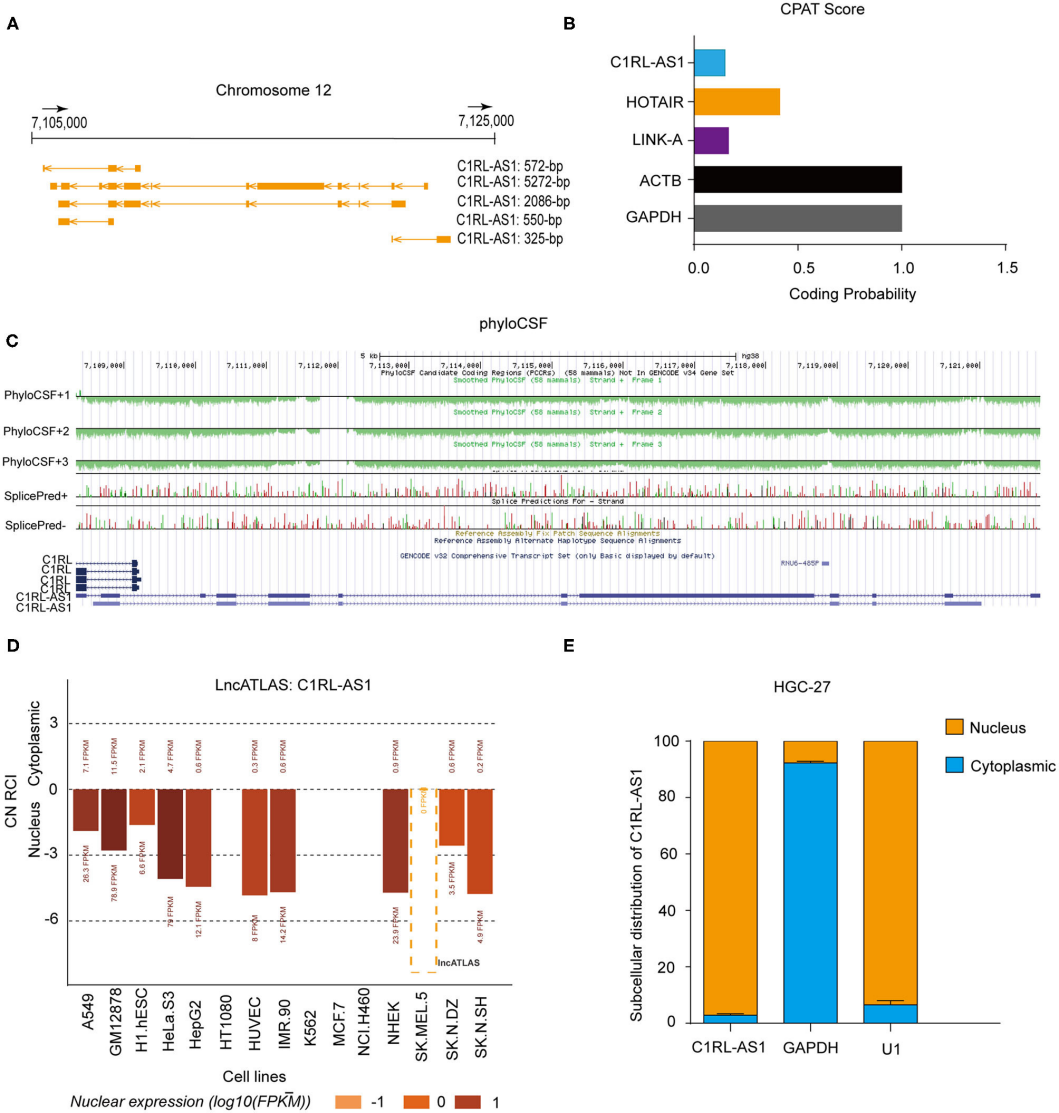
**(A)** Schematic representation of the genomic locus and isoforms of lncRNA C1RL-AS1 from the UCSC Genome browser (http://genome.ucsc.edu/). **(B)** The coding potential of C1RL-AS1 was analyzed using the Coding Potential Assessment Tool (CPAT). HOTAIR and LINK-A served as noncoding RNA controls, and ACTB and GAPDH served as coding RNA controls. **(C)** PhyloCSF codon substitution frequency analysis of C1RL-AS1. **(D)** The subcellular location of lncRNA C1RL-AS1 in 15 human cancer cell lines (http://lncatlas.crg.eu/). **(E)** The subcellular distribution of lncRNA C1RL-AS1 in HGC-27 cells (cytoplasmic, blue; nuclear, yellow). GAPDH served as the cytoplasmic internal control, and U1 served as the nuclear internal control.

LncRNA C1RL-AS1 is located on chromosome 12 and has five transcripts (Figure 2A). The results from the Coding Potential Assessment Tool (CPAT) and PhyloCSF codon substitution frequency analysis demonstrated that C1RL-AS1 is a noncoding RNA (Figures 2B,C). The results from LncATLAS (http://lncatlas.crg.eu/) revealed that C1RL-AS1 is mainly distributed in the nucleus (Figure 2D). Consistently, nuclear/cytoplasmic fractionation of HGC-27 confirmed that C1RL-AS1 is distributed in the nucleus (Figure 2E). Taken together, the aforementioned results suggest that C1RL-AS1 is mainly distributed in the nucleus and might play an oncogenic role in GC.

### Silencing LncRNA C1RL-AS1 Suppressed the Malignant Phenotype in GC

**Figure 3:**
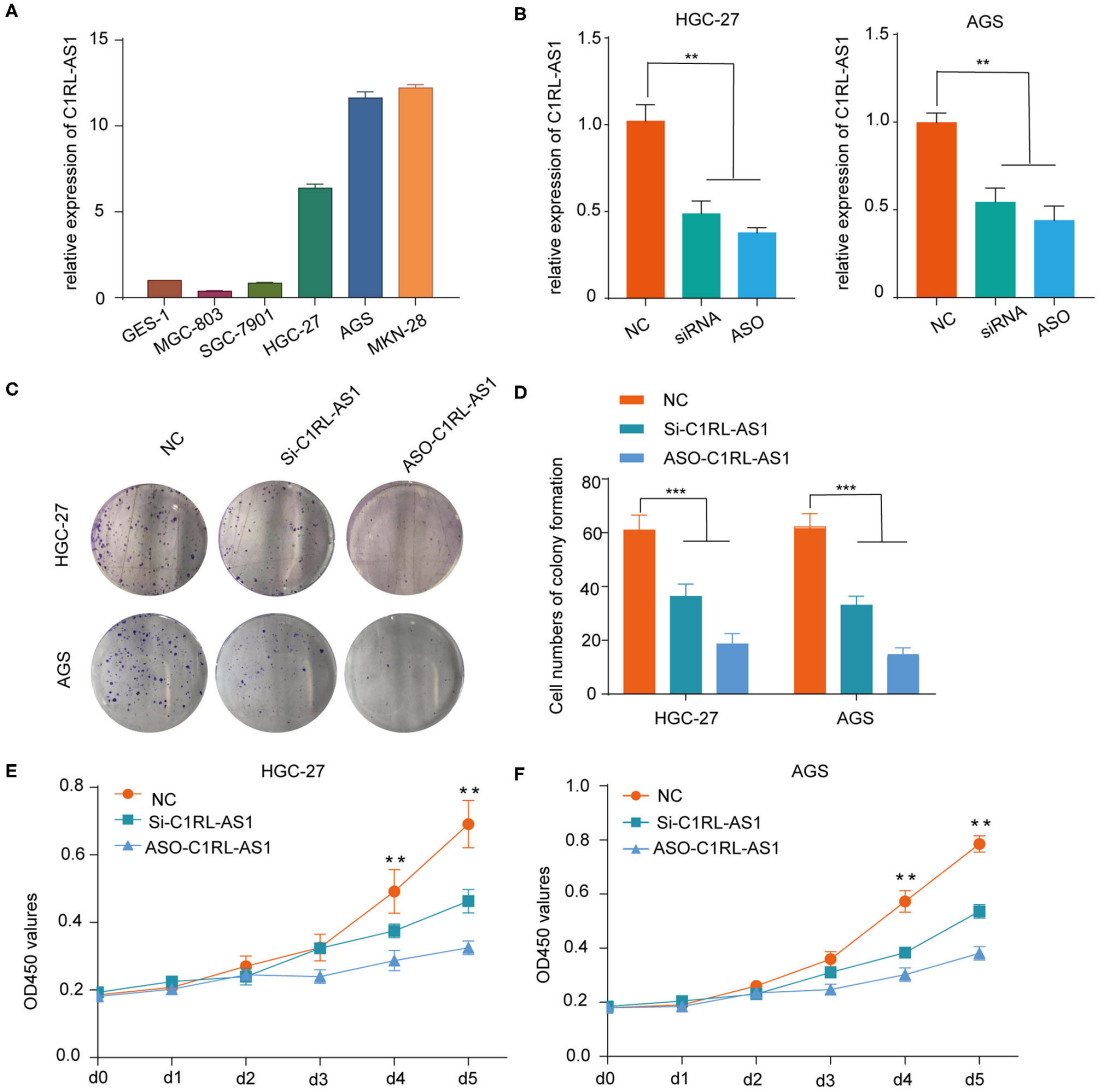
**(A)** Various levels of C1RL-AS1 expression in gastric cancer cell lines. **(B)** The silencing efficacy of siRNA and ASOs in HGC-27 and AGS cells was detected by qPCR. **(C,D)** Colony formation assays with representative images of HGC-27 and AGS cells after transfection with lncRNA C1RL-AS1 siRNA and ASO. **(E,F)** CCK-8 assays of HGC-27 and AGS cells after inhibiting C1RL-AS1 expression with siRNA and ASOs. Data are shown as the mean ± SEM, *n* = 3. *p < 0.05, **p < 0.01, ***p < 0.001.

First, various expression levels of C1RL-AS1 in GC cells and the knockdown efficiency of siRNA and ASOs were detected by qRT-PCR (Figures 3A,B). Then, *in vitro* functional assays were conducted in HGC-27 and AGS cells. As shown in Figures 3C,D, the colony formation results showed that the proliferation of HGC-27 and AGS cells was dramatically inhibited after silencing C1RL-AS1. Consistently, the CCK-8 assay also validated that silencing C1RL-AS1 weakened the proliferation of GC cells (Figures 3E,F).

**Figure 4:**
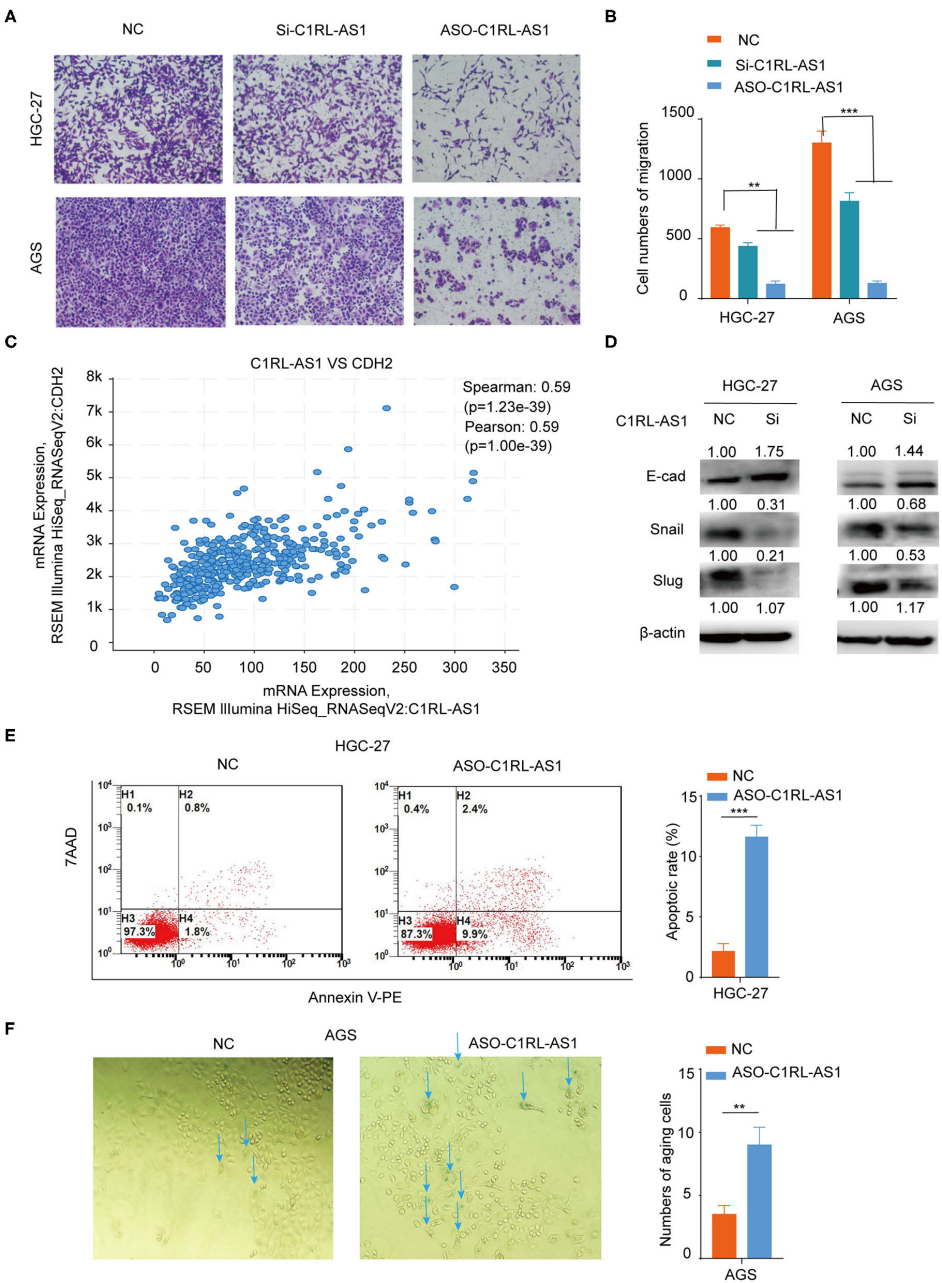
**(A,B)** Transwell migration assays of HGC-27 and AGS cells after silencing C1RL-AS1 expression. **(C)** The positive correlation expression between C1RL-AS1 and CDH2 (N-cadherin) in TCGA database. **(D)** Western blotting was applied to assess the expression levels of E-cad, Snail, and Slug after silencing C1RL-AS1 in HGC-27 and AGS cells. **(E)** The apoptotic rate of HGC-27 cells was detected by flow cytometry after transfection with C1RL-AS1 ASO. **(F)** The number of aging cells was calculated after transfection with ASO-C1RL-AS1 in AGS cells. Data are shown as the mean ± SEM, *n* = 3. **p < 0.01, ***p < 0.001.

Next, a transwell assay was used to assess the effect of C1RL-AS1 on migration. As shown in Figures 4A,B, the migration abilities of HCG-27 and AGS cells were strongly weakened after C1RL-AS1 expression was knocked down. Interestingly, the results from TCGA analysis showed that C1RL-AS1 had a positive correlation with CDH2 (N-cadherin) (Pearson: 0.59, *p* = 1.00e−39), suggesting the potential regulation of the EMT process (Figure 4C). Indeed, the expression of Snail and Slug was downregulated, whereas E-cadherin was upregulated after silencing C1RL-AS1 (Figure 4D). In addition, the apoptotic rate was significantly increased after silencing C1RL-AS1 by ASOs in HGC-27 cells (Figure 4E).

We also found that cellular senescence was enhanced when C1RL-AS1 expression was inhibited in AGS cells (Figure 4F). Moreover, p21 was found upregulated in both HGC-27 and AGS cells after knockdown C1RL-AS1; p53 was found elevated in AGS cells and no obvious changes of p16 were detected in neither cell line (Supplementary Figure 1). The aforementioned results suggest that C1RL-AS1 does have some impact on senescence.

Taken together, the results from colony formation, CCK-8, migration, apoptosis, and senescence assays suggested that C1RL-AS1 probably plays an oncogenic role and promotes a malignant phenotype in GC.

### Wnt/β-Catenin Was Involved in LncRNA C1RL-AS1-Mediated Pathways

**Figure 5:**
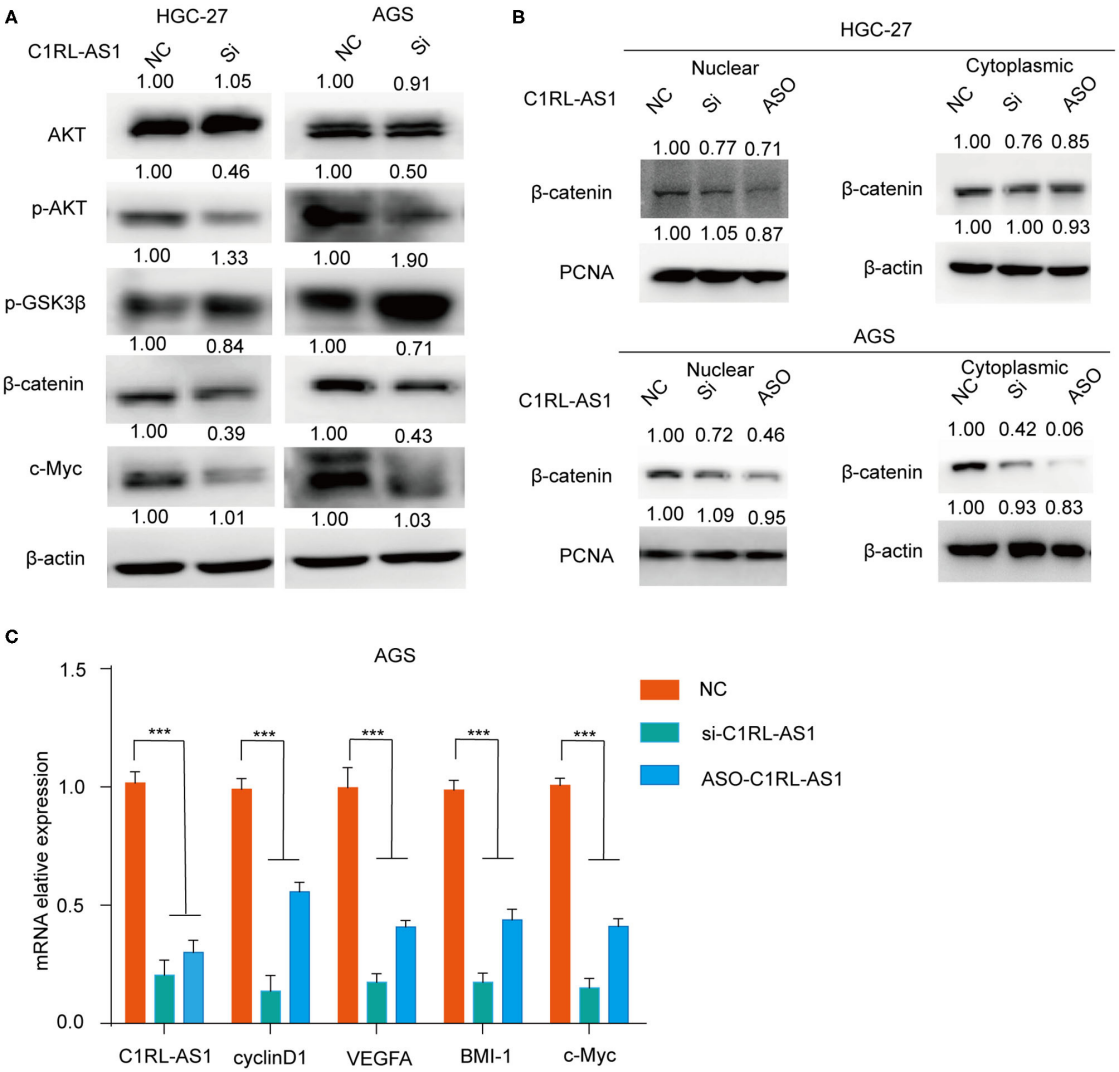
**(A)** The expression levels of AKT, p-AKT, p-GSK3β, and β-catenin in HGC-27 and AGS cells were detected by western blotting after silencing C1RL-AS1 with siRNA. **(B)** The expression of β-catenin in the cytoplasm and nucleus after inhibiting C1RL-AS1 by siRNA and ASO. β-Actin served as the cytoplasmic internal control, and PCNA served as the nuclear internal control. **(C)** The mRNA expression levels of C1RL-AS1, cyclin D1, VEGFA, Bmi-1, and c-Myc after silencing C1RL-AS1 expression in AGS cells. β-Actin served as the internal control. Data are shown as the mean ± SEM, *n* = 3. ***p < 0.001.

To investigate whether Wnt/β-catenin was involved in lncRNA C1RL-AS1-mediated pathways, western blotting was performed to detect the expression levels of key molecules in the pathway. As shown in Figure 5A, the expression levels of p-AKT and β-catenin were decreased, whereas p-GSK3β was increased after silencing C1RL-AS1 expression in both HGC-27 and AGS cells. To further confirm this, nuclear/cytoplasmic fractionation was carried out to examine β-catenin in the cytoplasm and nucleus. Unsurprisingly, β-catenin in the cytoplasm and nucleus was decreased after C1RL-AS1 expression was knocked down (Figure 5B). Moreover, the downstream targets of the β-catenin pathway were detected by qPCR. As shown in Figure 5C, downstream targets such as cyclin D1, BMI-1, c-Myc, and VEGFA were significantly downregulated after silencing C1RL-AS1 with siRNA and ASOs. All the aforementioned results suggested that C1RL-AS1 probably exerts its function through the Wnt/β-catenin pathway.

### LncRNA C1RL-AS1 Mediated β-Catenin Pathway by Modulating AKT

**Figure 6:**
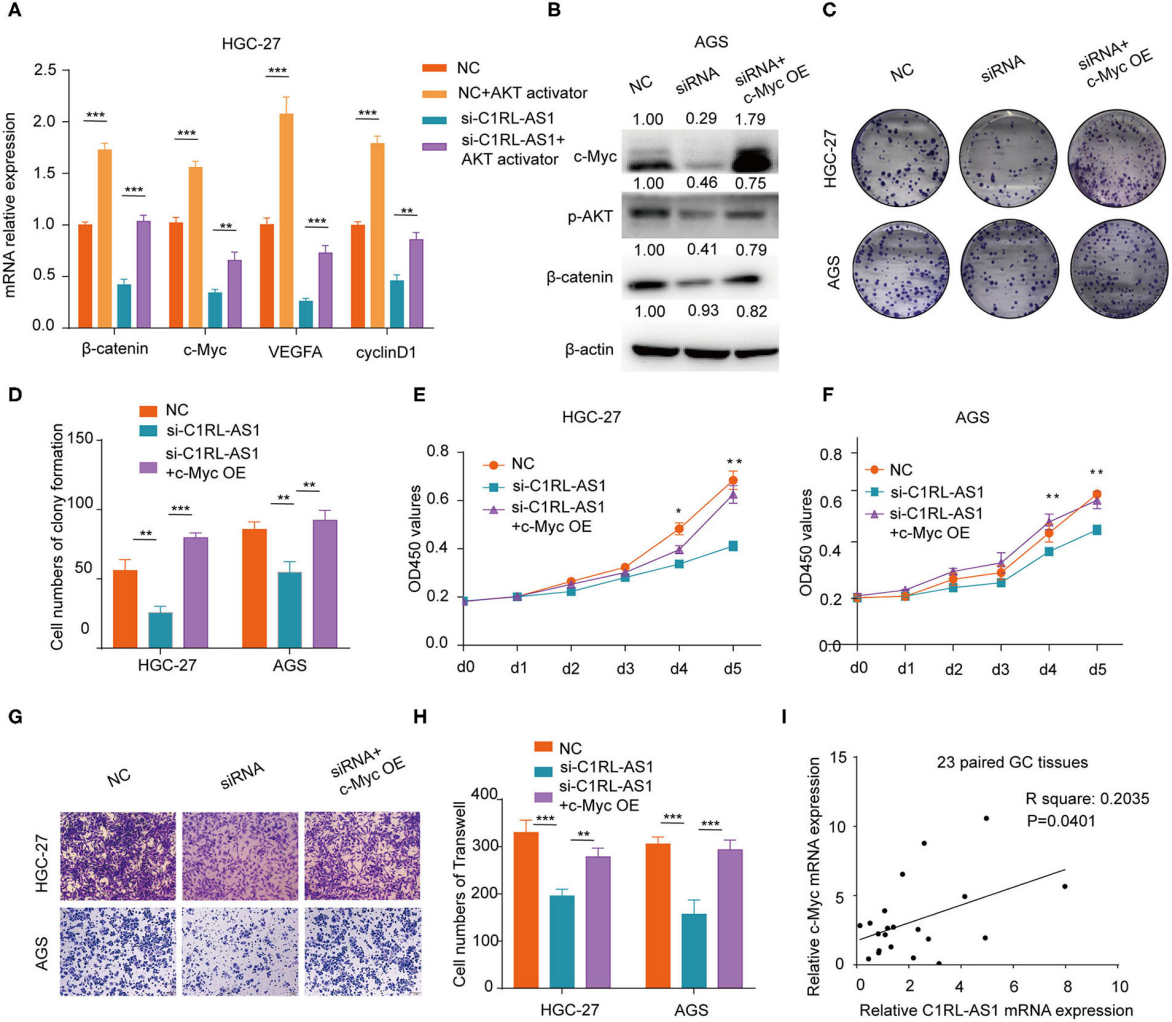
**(A)** Quantification of β-catenin, c-Myc, VEGFA, and cyclin D1 mRNA levels by qPCR after the indicated treatment in HGC-27 cells. **(B)** Western blot of the protein levels of c-Myc, p-AKT, and β-catenin after overexpressing c-Myc in AGS cells after silencing C1RL-AS1 expression. **(C,D)** Colony formation of C1RL-AS1-silenced HGC-27/AGS cells after overexpressing c-Myc. **(E,F)** CCK-8 assay was used to assess the proliferation of C1RL-AS1 silenced HGC-27/AGS cells after overexpressing c-Myc. **(G,H)** Transwell assays were used to assess the migration abilities after overexpressing c-Myc in C1RL-AS1-silenced HGC-27/AGS cells. **(I)** The correlation between C1RL-AS1 and c-Myc expression in 23 paired GC patients. Data are shown as the mean ± SEM, *n* = 3. *p < 0.05, **p < 0.01, ***p < 0.001.

Numerous studies have demonstrated that AKT is a key node modulator in diverse pathways. To verify whether lncRNA C1RL-AS1 was dependent on AKT to mediate the β-catenin pathway, rescue experiments were conducted. First, an AKT activator, SC-79, was added to C1RL-AS1-silenced cells to overexpress AKT. As shown in Figure 6A, the mRNA levels of target genes involved in the β-catenin pathway, such as β-catenin, c-Myc, BMI-1, and cyclin D1, were upregulated after SC-79 treatment and restored to the parental level after silencing C1RL-AS1 expression. This suggested that C1RL-AS1 probably activates the Wnt/β-catenin pathway by upregulating AKT.

### LncRNA C1RL-AS1 Was Dependent on c-Myc to Exert Diverse Functions in GC Cells

**Figure 7:**
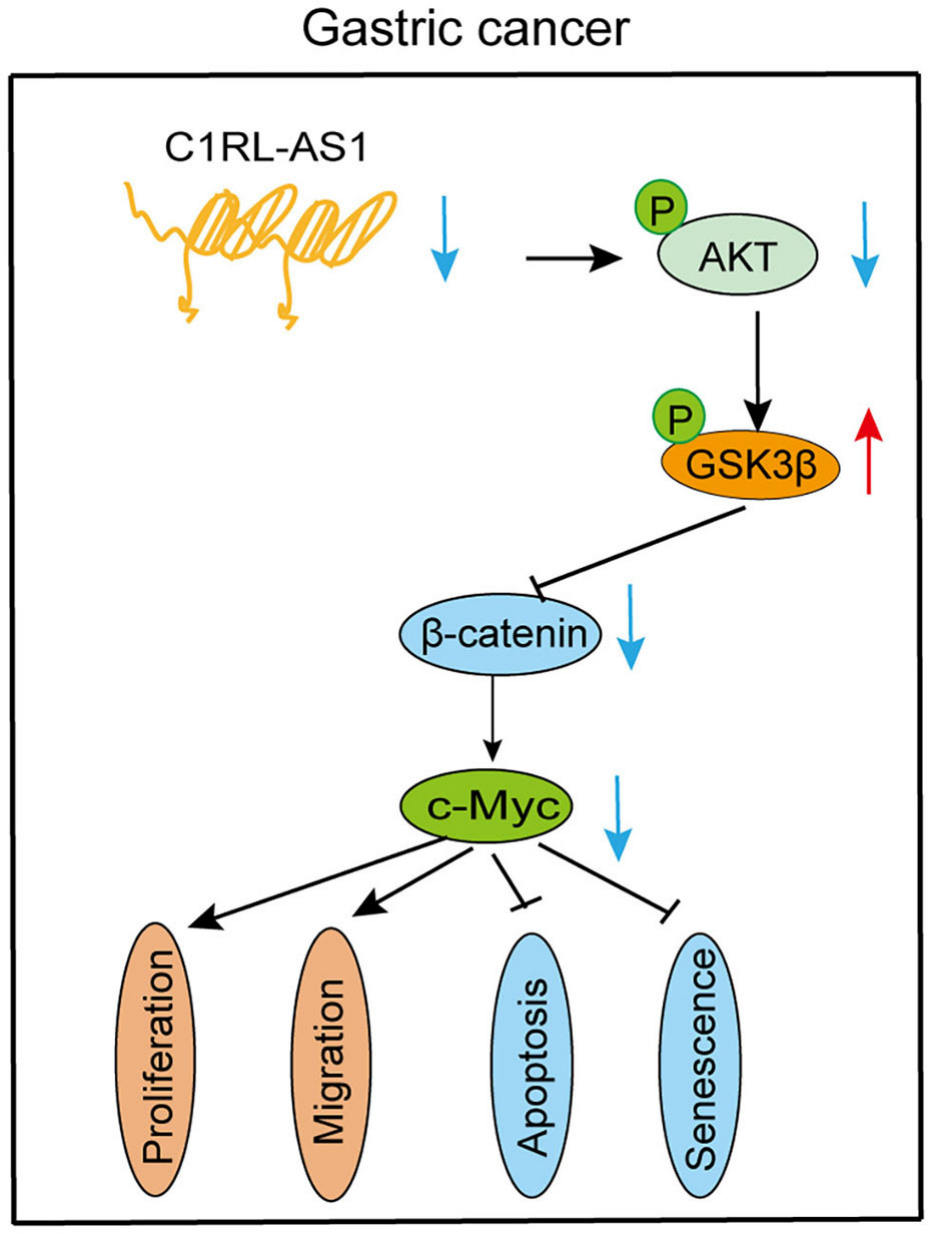
Diagram of the biological mechanism of lncRNA C1RL-AS1 in GC.

c-Myc, one of the downstream targets of the AKT/β-catenin pathway, is reported to promote malignant behavior in various cancers. Next, the c-Myc overexpression plasmid was transfected into C1RL-AS1-silenced cells. As western blotting revealed in Figure 6B, the expression levels of p-AKT and β-catenin were restored to the parental level. Moreover, colony formation, proliferation, and migration abilities were restored to the parental level (Figures 6C–H). In addition, the results of qPCR in the 23 paired GC and normal tissues showed a positive correlation between C1RL-AS1 and c-Myc expression (Figure 6I, *R*^2^: 0.2035, *p* = 0.0401). All the aforementioned results indicated that lncRNA C1RL-AS1 probably exerts its function through the AKT/β-catenin pathway by upregulating c-Myc (Figure 7).

## Discussion

GC remains a common malignant tumor worldwide. However, the investigation of lncRNAs in the development and progression of GC is limited. In the current study, a novel lncRNA termed C1RL-AS1 was identified and found to be significantly elevated in the analysis of both the TCGA and our FUSCC database. Moreover, high expression of C1RL-AS1 was correlated with short DFS and OS times. Subsequent *in vitro* experiments showed that C1RL-AS promoted proliferation and migration and inhibited apoptosis and senescence in GC cells, indicating an oncogenic role in GC. The underlying mechanism suggested that Wnt/β-catenin is involved in C1RL-AS1-mediated pathways. Further, C1RL-AS1 probably exerts its biological function through the AKT/β-catenin pathway by upregulating c-Myc, suggesting a potential therapeutic target in GC.

Previous investigations have shown that lncRNAs exert their biological functions through diverse mechanisms. They can serve as signals to promote transcription, as baits to suppress transcription, or as epigenetic regulators (18, 19). They can also act as scaffolds to interact with various proteins to form RNA/protein complexes to exhibit diverse regulatory mechanisms (20). Nevertheless, mounting evidence indicates that lncRNAs are involved in the transduction process of various signal pathways (21). For example, lncRNA TSLNC8 acts as a tumor suppressor in hepatocellular carcinoma and exerts its effects through the JAK/STAT3 pathway (22). LncRNA AK023391 promotes proliferation and migration in GC through the PI3K/AKT pathway (23). In this study, we found that β-catenin expression levels were decreased in both the cytoplasm and the nucleus after silencing C1RL-AS1. In addition, downstream targets of the β-catenin pathway, such as VEGFA, Bmi-1, and c-Myc, were downregulated, suggesting the involvement of Wnt/β-catenin in lncRNA C1RL-AS1-mediated pathways.

The Wnt/β-catenin pathway is usually activated in many types of cancer and participates in a diversity of biological processes, especially the regulation of EMT. Recently, several studies have shown the role of lncRNAs in the regulation of the Wnt/β-catenin pathway. Xu et al. found that silencing the expression of lncRNA ZFAS1 inhibited the malignancy of GC cells by blocking the Wnt/β-catenin pathway (24). LncRNA SNHG20 was also demonstrated to promote GC progression through the GSK3β/β-catenin pathway by regulating p21 expression (25). AKT acts as a key node modulator in diverse pathways and affects downstream GSK3β expression to activate the Wnt/β-catenin pathway. Here, through SC-79 (an AKT activator) treatment and rescue experiments, we found that C1RL-AS1 probably activates the Wnt/β-catenin pathway by upregulating AKT.

C-Myc, which serves as one of the downstream targets of the β-catenin pathway, has been reported to promote proliferation and migration and maintain stemness in various cancers (26, 27). Here, both the mRNA and protein levels of c-Myc were downregulated after C1RL-AS1 expression was knocked down. In addition, the results of the rescue experiments on proliferation and migration suggest that C1RL-AS1 is probably dependent on c-Myc expression to exert its function. Moreover, C1RL-AS1 and c-Myc expression at the mRNA level in 23 paired GC patients displayed a positive correlation. Together, these results indicate that C1RL-AS1 probably promotes a malignant phenotype through the AKT/GSK3β/β-catenin pathway by upregulating c-Myc.

Collectively, our study revealed that the C1RL-AS1/AKT/GSK3β/β-catenin/c-Myc pathway promotes a malignant phenotype in GC, indicating that it might be an attractive therapeutic target for GC patients in the future.

## Data Availability Statement

The raw data supporting the conclusions of this article will be made available by the authors, without undue reservation, to any qualified researcher.

## Ethics Statement

The studies involving human participants were reviewed and approved by the ethics committee of Fudan University Shanghai Cancer Center. Written informed consent to participate in this study was provided by the patients.

## Author Contributions

GW-J designed and supervised the study and helped write, review, and edit the article. GY-W conducted most of the experiments, and WZ-H drafted the article. ZJ-Y and GZ contributed to the collection of GC samples, and ZL-Q helped perform the data analysis. All the authors read and approved the final article.

## Conflict of Interest

The authors declare that the research was conducted in the absence of any commercial or financial relationships that could be construed as a potential conflict of interest.

## Supplementary Material

Supplementary Figure 1 The expression levels of p16, p21, and p53 in HGC-27 and AGS cells were detected by western blotting after silencing C1RL-AS1. GAPDH served as the internal control.
