# Supplementary figures and images for: Silencing of LncRNA C1RL-AS1 Suppresses the Malignant Phenotype in Gastric Cancer Cells via the AKT/β-Catenin/c-Myc Pathway
Source: Front Oncol. 2020 Sep 2;10:1508. doi: 10.3389/fonc.2020.01508 (PMC7492601; doi:10.3389/fonc.2020.01508)

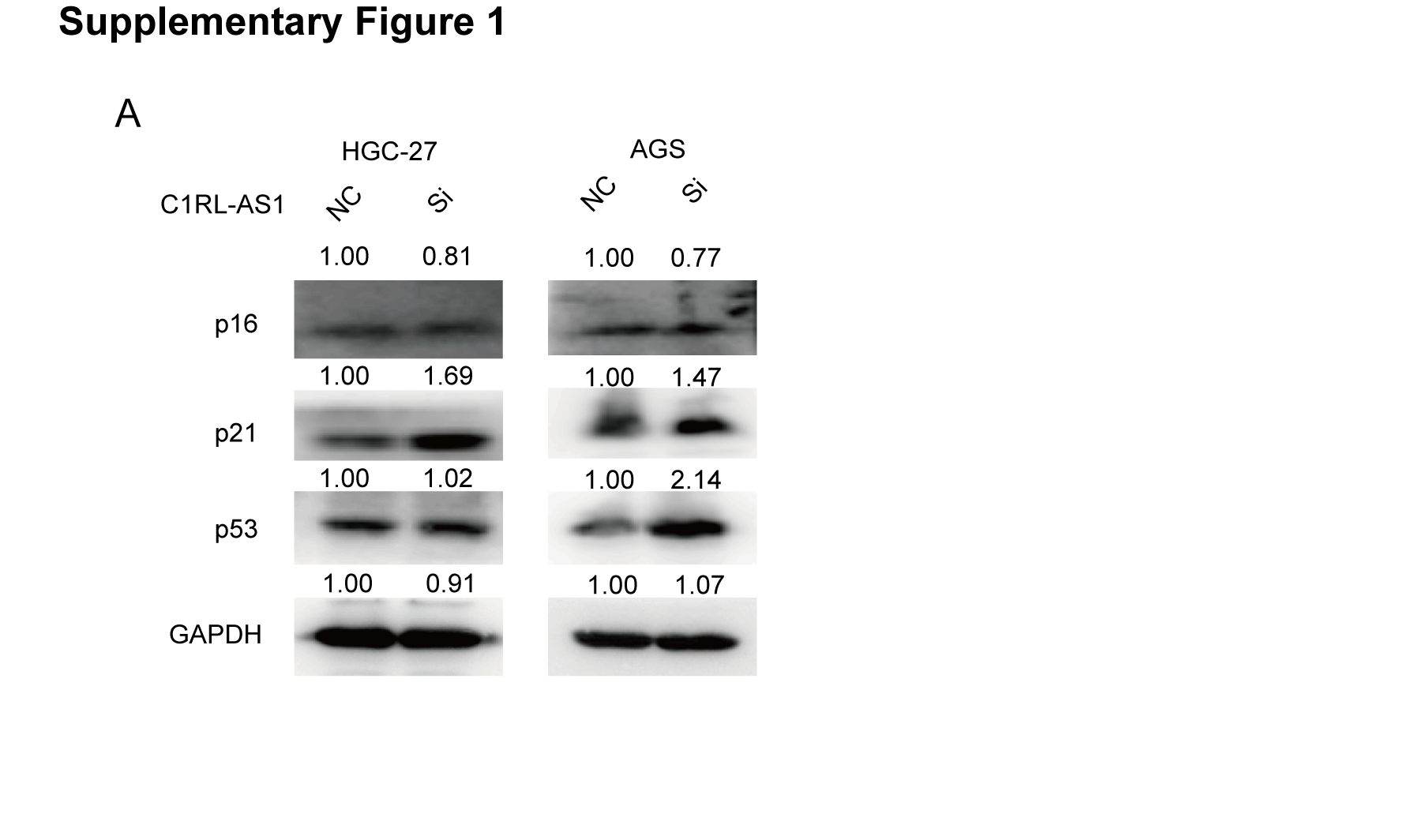

Supplement: Supplementary Figure 1 — The expression levels of p16, p21, and p53 in HGC-27 and AGS cells were detected by western blotting after silencing C1RL-AS1. GAPDH served as the internal control. [file Image_1.TIF]
