# Supplementary material for: Silencing of LncRNA C1RL-AS1 Suppresses the Malignant Phenotype in Gastric Cancer Cells via the AKT/β-Catenin/c-Myc Pathway
Source: Front Oncol. 2020 Sep 2;10:1508. doi: 10.3389/fonc.2020.01508 (PMC7492601; doi:10.3389/fonc.2020.01508)
Supplement: Supplementary file 2 [file Data_Sheet_1.docx]

**Supplementary materials and methods**

1. **LncRNA C1RL-AS1(ENST00000541775.5_1) sequence:**

GAGCCTCACAGCAAAGCCCTCTGGAGCCTTGATGTCCGTGCTGCTCTCTTGGCCTTTGCCATACGGCTCTGGGTACCCGGGGGATGTCAGCTGCTGGGGTAGCTCTTGGGCCAAGAGGACGGAGCCCCGGGTTGGGCAAGCCTGGAGGACTCCCCAGAGAAGCAGCCACCACCTCCTCCCGCGCAGCCTGAGCTCTAGGATTGTGGGTTTTCCATCCTCCGAAGACATCACCTTTGCTCATCTCCCAGGAGAGTCTCGTCCAAAGGAGGGGGGTGCTTTCTGCTTCAGCAGATCCCACCCCACCCTGGGATCCGAGGGAGCAATGGTGGGGCGAGTGAGGGTCTGCCGTAAATATCCCCCGACCACCCTCTGGGAAGGTGCTAGAGGCCACAGGCAAATTTCAGTCTCCCCATGGAATATCTGCTGTGCTGCTGCTGCTGCTGCTGCTGGCTAGAGAGATTTAGGAGAGCCACAGTCTATGGCTACAAGGAGATGATAAGAGAGTGATGGCCAGGACAGATGCAATGTCTCAGAACTCTTAGGGTCACCTGGAGCACCTTGGGAGCCAGAGATGCAGCCAAAGAGTGAACAATGGTACCAATCCCAAGACCCATGTGTTAAGAAGAAACCAGCCAAGACTCAGATCAGGATTGGGGAGTCCACCCCTCGTTTCCCTTCAGAGCATTATTTCCTTGTTTGCTAAGAGGGGCAGGAACTGGAAAACTCAGGCTAGCCCATCTGAGAAGACTTTCCTGGAGATGAGCTGGAGGAGGGAGGAGTAAGGCCTTGGAAGGAAACTCCAGGACAGCACCACCTCCCCTACACTGGGACCTCGTCTCTAACTGGCAGAGTGGTCAATGAAAAAAAGGGCATGCCTTGGCACATCATGGTCAAACTGCTAAAAGCCAAAGACAAGAGAAAATCTTTTCTTTTTTTTAATCCCATCCTAAAGACTAGACAGAACAATATTTTCAATGTCACTCTTTTCTCATCAAAACCAATATAGACCAGAAGACAGTGGAATATCTCTAAAATGCTGAAGGAAAAAAAAAGTCAACTCAGAATTGTTTTTCTAGTGAAAGTATTCTTCCAGAATGAAGATGAAATAAAGCTATTTTCAGATAAAAGAAAACAGAGATTCTTGTCGCCAGCAGACCTCCACTATGAGAAGCGCTAAAGAAAGTTCTTTAGGACGGGCACGATAGCTCACGCCTATAATCCCAGCATTTTGGGAGGCCAAGGTGGGAGGATTGCTTGAGCTCGGAAGATGGAGGCTGCAGTGAGCCATGACGGTGCCACTGCACTCCAGCTTGGGTGACAGAGTGAGACGCTGTCTCAAATTGGAAGAGCAGAGCTGCAGACAGGAATAAGAAGAACGGTAAATTTGAAGATGGGACCGTCTAGTTGCAAGAAAACAAGCTCAGGGCTCCCACTGATTCTACATTACGGGCTGTGGGTTCCTTGAGGGAGGAATTTTGTCCTGCAGGCTGTGGCAGCCCTAGGACTCAGCACATCTAGGTGTCCACCTGCGTCTGTGGTGAGAAGCCTGATCATTGCCACGTGACCGCCTCCTCACTGTTGGTACCTGGTTGTAGAGAGCAGCTCTGTGAGTAGAAGGAGAAGGATCCTGCCCTGGAGAGGGCCTGGCTGGTCCTCACCTGGGGCCTATCATTTAGTAACAGGGCTTCTGGAAGTGGTAGCAGATCTGGGCCTGGTGGGCATTCTGCTTCTCCTGGGTGGCAGCAGAAGTCCCTAGGTGGCAGGCTGGGGACTGCCCAAAGGTTCTGACTCAGGAAGAGCACAGTGGAACAGAAAGCCCTTGTACCACCTTCGCTGCTGTATTCGTCCATTCTCACACTGCTGTACAGAACTACCTGAGACTGGGGAATTCATGAAGAGAAGAAGTTTAATTGACTCACAGTTCTGCGGGCTTAACAGGAAGCATGACTGGGAGGCCTCAGGAAGCTTATAATCATGGCAGAAGGCGAAGGGGAAGCAAGGACCTTCTTCACATGGCAGCAGGAGAAAGAGAAGAAGGGAGAAGTCTACACACTTTTAAACAACCAGATCTCATGAGAATTCCATCGGGAGACAGCACTAGGGGGATGGCACTAAACCATTAGAAACTGCCCCCATGATCCAATCACCTCTCACCAGGCCCCTCCTCCAACACGTGGGGATTACAATTCCACATGAGATTTGGTTGGGGACATAGAGCCAAATCCTATCAGCTGGTATCCTCCTTTTTCCAGGCTTTAGATATGGCACAACCTGATGGCTTCTGCATAAAGCCTCCTCTCATCTGCATCTCTCCACCTCACTCACCCTCTTTTCAGCAAAGAACCCTCTGGCTCTAGTTTCACTGTGGTGGTCCTGGGAGAGGTAGAGAAGACCCAGAGGCTCCTTACCCTGGGAGGTCAAACTGACCATCCAGGTTTGGCTCTTCCCTGCTGGTGTGTCTAGAACACCATCTTCCCCTTATTGCTGTGGACTTCAGGGCTCTGCTGCTCTGTCCTCCACAGAACTTGTCAATCAGCTTCCTACCAGCTAAGATCTGGTGCTGTCTGATATATCCTCCCTGCGGCTTGTCACTCATGCTGTCTAATGGCGTGAGGTGCATTGAGTTGTATTCTGATGTCTTTGTCCTTTTGAAGCCACAGAGTAAGGTCACACAACTTTGAAACGAGCAGGGTACCATCTGAAGATGCTGGACATTCCTTTTAACAATTACCTAGGGGAGAGGCAGTCCTCAGACAGGACTCAGGTGGGTGGGCCTCAAAGAGGAAGGGAAAGGGGGCAGCTGCCTGCCTCTGAACTTCACTCTCTAGACTCGTGGAAGGGTCTAGCTTAGAGCTAGGATGGGAGGGCTGTGAGCACTGAGGATGGAGTCATGCCTTTTTGTGACTGGACACATTTTTACTGCCCGAAGTCATCTACTGCCCCCACCCCCCAACCCCATTCTTCTGCCCACTTAGCGAATATTGAGGTTATGGTCAGGATGGGGTCATTTGCAATGAAATTCAAGGTTGAGACTATGTTGCAGTTATCTGTCTGCTTGTAAAAAGTCACCCCCACATTTTGTTTAAAACAGCAAGTATGTACTATTTCACACAGTTTCTGTGGGTCAGGAATCCAGAAGCGGCTGAGATGGGTAGTTTAGGATCAGGGTCTCTCACAAGGCTTTGGTCCAGGTGCAGGCTGCAGTCCTCCAGAGGCGGGACTGGGGCTGGAGGTCTGCTTCCAGACGGTCACTCACATGGATGTGGGCAGGCGGCCTCAGTTCCCTGCCACATGGCCCTCCTCATAGGACGCTTGAGTGTCCTCGCGGCATAGCAGCTGGCTTTCCCCAGAGTGACCCAAGGTGGAAAGAACAAGGCAGAGGTGACAGTGTCTTTTATAAGCCAGCCTTGGAAGTGACGTAGCATCATTTCTGCCTTATTCCATTGGTCACACAGACAACCCGATGTGGAAGGAGACATCACAAGGACAGGAATACTGGGAAGCAGGTATTGTTGGTGGCTGTCTCGGACGCTGGTTCTCATAGAAGTAGATGTATTATTTTAGTACAATAAGTGCAGACGATCAGCTAGCAGGATGTTATTTTTGCTGGGTATGGTTGTATTTCAGGTGCATTTACCCAAATCCAGAGTACCTTTGGGGGCTCTGACCCTCTCCTCCTCCACCTTGAGATCTGTAGAGGCCTCTCGTTCATCCCACCAAGAGCAGGACTGGACAGATGAAAGCATGGCTTACAGGAGGCCAACACAATAGGAGGTAGGGCTTTGGGAAGACAGCATAGGTTTCAAAAGAACCAAGAAGCTGTTGAAAAGGAGAGCAGATGGGCAGGGAAGACAAGTCCCAGATAGAATGCCCTTTGCAACCTAGGGGGAGCTGACCACAGTGTGCATCCTGACACTCGAAGACAAGGTCTCCATCAGCAGATTGCCCAACAGAACAAGTTCAGAGCCCAGAGATCCCAGTTCCAGATCTCAGATCTCCCACACCCTGTGGGCTCTGTGTGGACTTTCGTCCTGAAACAAAGTCCTTTAGTAAAGGACTCTTTAAATTCACTCCATCTGTGCTTTGCTTGCCAATAGCAGGGTGCGTCTGTTACATACTGGACTATCCCTTCCTTCAATATGTTCATGGCTGAGGCTGCATTTGTGCCTCAGATAATCAAGGTGAGTATAACTCTCTGTTCTGTTTCCCTTTTGTTTCCTCCAAGACTTTAATAAACCCTCATTCAAGAGGCCCACCTTGTCCCACCTGTGCTTCTCAGAAAAGAAGGAGTGGTGGGGAGACAGGCTGGGGTGGGGGCTTTGCCACCTGGCTGCCCCTTCACCTGTGGGTTCTTGCTCCAATGAGGACCCAGCAGGATAAGCTTTAGTTTCATTAAGGGTGACAATAGTTTCCTTACAGCTGGGTCAAGGATATGAGCGAGCCTCTTCCGAAAACAGCCGGGAAGGGAGAGGAATCCAAGAGGAGGAGCAGGTGGGAAAGACAAGACAGAAGGTGGATCGGGACCAGGCCTGGGAGGGAGCACAGGCACACAGAAGTGAAAGAATTCGCCTGAGGTTACACGACTCCTCGAGCGGTGCCTTTAGCTCTGCATTGTGCCACCTGCCTGGGTCAAGGAAGATGCCACCGCTGTTGTTCTGTGTACAGAGAGGGAAAGGAAGATGCTGGCTCTCAGCAGTTCTATAGAACAGAGGATGGAGAGGCCACACGACAATAGGCACACGCACCGTACATTGAAGAAGTGCATGCAAGACAGAATCGTGTGGGATATGCACACTTGCGTGGAAGAAAAACTGTTCCATTCACATTGAGAAGAAGAAGAAGAAGAGGAGGAGGAGGAGGAGGAAGAAGAAAGATGGTGAGATATCCTGCAGGAGACCAGGGCCAGGAGGGTGGAGGCCTCTCAAGAAGAGGGAAGCATGAGGAAGCTCCAGGGCAGGATGGTTTACCTGCCTGGACAGCAAGATGATGGCTACACTAGCCCCCATTCTCTGGGCGCCTGGATTTGCCCACCAGATCTCCTCACCTCTTGCCCTTCACCTCCTGCTGTACCTACAAGGTCTCCCCGATTCTCATCTGCCCATAATCATGGACACAGCCCCAGGATGTGCAGGACTCTCAGGGACCATCTGGAGTTCCAGCTGGAATCTGGGCCTGGTGGAGTGGGAGTGGGGCAGGGGCCTGCATTGGGCTGACTTAGAGAGCACAGTTATTCCATCCATATGGAAATAAACATTTTGGATTCCTGATCACA

Yellow: siRNA target sequence; Green: ASO target sequence;

1. **Information and cDNA sequence of pENTER vector**
2. the map of pENTER vector

**
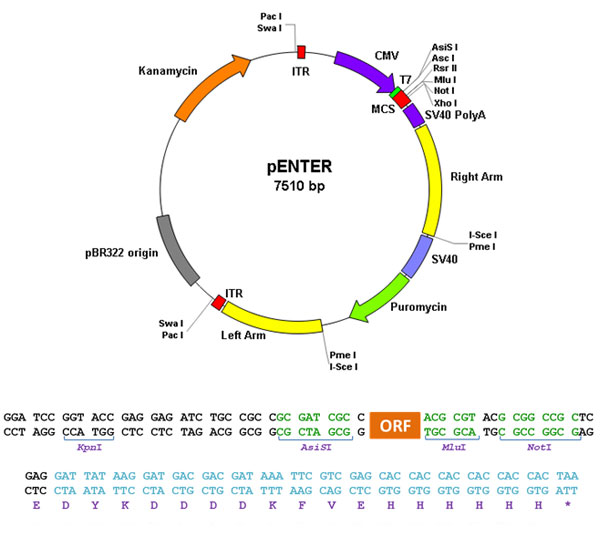
**

1. cDNA sequence of pENTER vector

TTCGAATTTAAATTAATTAACATCATCAATAATATACCTTATTTTGGATTGAAGCCAATATGATAATGAGGGGGTGGAGTTTGTGACGTGGCGCGGGGCGTGGGAACGGGGCGGGTGACGTAGTAGTGTGGCGGAAGTGTGATGTTGCAAGTGTGGCGGAACACATGTAAGCGACGGATGTGGCAAAAGTGACGTTTTTGGTGTGCGCCGGTGTACACAGGAAGTGACAATTTTCGCGCGGTTTTAGGCGGATGTTGTAGTAAATTTGGGCGTAACCGAGTAAGATTTGGCCATTTTCGCGGGAAAACTGAATAAGAGGAAGTGAAATCTGAATAATTTTGTGTTACTCATAGCGCGTAATACTGTAATAGTAATCAATTACGGGGTCATTAGTTCATAGCCCATATATGGAGTTCCGCGTTACATAACTTACGGTAAATGGCCCGCCTGGCTGACCGCCCAACGACCCCCGCCCATTGACGTCAATAATGACGTATGTTCCCATAGTAACGCCAATAGGGACTTTCCATTGACGTCAATGGGTGGAGTATTTACGGTAAACTGCCCACTTGGCAGTACATCAAGTGTATCATATGCCAAGTACGCCCCCTATTGACGTCAATGACGGTAAATGGCCCGCCTGGCATTATGCCCAGTACATGACCTTATGGGACTTTCCTACTTGGCAGTACATCTACGTATTAGTCATCGCTATTACCATGGTGATGCGGTTTTGGCAGTACATCAATGGGCGTGGATAGCGGTTTGACTCACGGGGATTTCCAAGTCTCCACCCCATTGACGTCAATGGGAGTTTGTTTTGGCACCAAAATCAACGGGACTTTCCAAAATGTCGTAACAACTCCGCCCCATTGACGCAAATGGGCGGTAGGCGTGTACGGTGGGAGGTCTATATAAGCAGAGCTGGTTTAGTGAACCGTCAGATCCGCTAGTAATACGACTCACTATAGGGAGAGGATCCGGTACCGAGGAGATCTGCCGCCGCGATCGCCGGCGCGCCAGATCTCAAGCTTAACTAGCTAGCGGACCGACGCGTACGCGGCCGCTCGAGGATTATAAGGATGACGACGATAAATTCGTCGAGCACCACCACCACCACCACTAATAAGGTTTATCCGATCCACCGGATCTAGATAAGATATCCGATCCACCGGATCTAGATAACTGATCATAATCAGCCATACCACATTTGTAGAGGTTTTACTTGCTTTAAAAAACCTCCCACACCTCCCCCTGAACCTGAAACATAAAATGAATGCAATTGTTGTTGTTAACTTGTTTATTGCAGCTTATAATGGTTACAAATAAAGCAATAGCATCACAAATTTCACAAATAAAGCATTTTTTTCACTGCATTCTAGTTGTGGTTTGTCCAAACTCATCAATGTATCTTAACGCGGATCTGGGCGTGGTTAAGGGTGGGAAAGAATATATAAGGTGGGGGTCTTATGTAGTTTTGTATCTGTTTTGCAGCAGCCGCCGCCGCCATGAGCACCAACTCGTTTGATGGAAGCATTGTGAGCTCATATTTGACAACGCGCATGCCCCCATGGGCCGGGGTGCGTCAGAATGTGATGGGCTCCAGCATTGATGGTCGCCCCGTCCTGCCCGCAAACTCTACTACCTTGACCTACGAGACCGTGTCTGGAACGCCGTTGGAGACTGCAGCCTCCGCCGCCGCTTCAGCCGCTGCAGCCACCGCCCGCGGGATTGTGACTGACTTTGCTTTCCTGAGCCCGCTTGCAAGCAGTGCAGCTTCCCGTTCATCCGCCCGCGATGACAAGTTGACGGCTCTTTTGGCACAATTGGATTCTTTGACCCGGGAACTTAATGTCGTTTCTCAGCAGCTGTTGGATCTGCGCCAGCAGGTTTCTGCCCTGAAGGCTTCCTCCCCTCCCAATGCGGTTTAAAACATAAATAAAAAACCAGACTCTGTTTGGATTTGGATCAAGCAAGTGTCTTGCTGTCTTTATTTAGGGGTTTTGCGCGCGCGGTAGGCCCGGGACCAGCGGTCTCGGTCGTTGAGGGTCCTGTGTATTTTTTCCAGGACGTGGTAAAGGTGACTCTGGATGTTCAGATACATGGGCATAAGCCCGTCTCTGGGGTGGAGGTAGCACCACTGCAGAGCTTCATGCTGCGGGGTGGTGTTGTAGATGATCCAGTCGTAGCAGGAGCGCTGGGCGTGGTGCCTAAAAATGTCTTTCAGTAGCAAGCTGATTGCCAGGGGCAGGCCCTTGGTGTAAGTGTTTACAAAGCGGTTAAGCTGGGATGGGTGCATACGTGTTTAAACTAGGGATAACAGGGTAATCGGCTGTGGAATGTGTGTCAGTTAGGGTGTGGAAAGTCCCCAGGCTCCCCAGCAGGCAGAAGTATGCAAAGCATGCATCTCAATTAGTCAGCAACCAGGTGTGGAAAGTCCCCAGGCTCCCCAGCAGGCAGAAGTATGCAAAGCATGCATCTCAATTAGTCAGCAACCATAGTCCCGCCCCTAACTCCGCCCATCCCGCCCCTAACTCCGCCCAGTTCCGCCCATTCTCCGCCCCATGGCTGACTAATTTTTTTTATTTATGCAGAGGCCGAGGCCGCCTCGGCCTCTGAGCTATTCCAGAAGTAGTGAGGAGGCTTTTTTGGAGGCCTAGGCTTTTGCAAAAAGCTCCCGGGAGCTTGTATATCCATTTTCGGATCTGATCAAGAGACACGTACGACCATGACCGAGTACAAGCCCACGGTGCGCCTCGCCACCCGCGACGACGTCCCCCGGGCAGTACGCACCCTCGCCGCCGCGTTCGCCGACTACCCCGCCACGCGCCACACCGTCGATCCAGACCGCCACATCGAGCGGGTCACCGAGCTGCAAGAACTCTTCCTCACGCGCGTCGGGCTCGACATCGGCAAGGTGTGGGTCGCGGACGACGGCGCCGCGGTGGCGGTCTGGACCACGCCGGAGAGCGTCGAAGCGGGGGCGGTGTTCGCCGAGATCGGCCCGCGCATGGCCGAGTTGAGCGGTTCCCGGCTGGCCGCGCAGCAACAGATGGAAGGCCTCCTGGCGCCGCACCGGCCCAAGGAGCCCGCGTGGTTCCTGGCCACCGTCGGCGTCTCGCCCGACCACCAGGGCAAGGGTCTGGGCAGCGCCGTCGTGCTCCCCGGAGTGGAGGCGGCCGAGCGCGCCGGGGTGCCCGCCTTCCTGGAGACCTCCGCGCCCCGCAACCTCCCCTTCTACGAGCGGCTCGGCTTCACCGTCACCGCCGACGTCGAGGTGCCCGAAGGACCGCGCACCTGGTGCATGACCCGCAAGCCCGGTGCCTGACTGTGCCTTCTAGTTGCCAGCCATCTGTTGTTTGCCCCTCCCCCGTGCCTTCCTTGACCCTGGAAGGTGCCACTCCCACTGTCCTTTCCTAATAAAATGAGGAAATTGCATCGCATTGTCTGAGTAGGTGTCATTCTATTCTGGGGGGTGGGGTGGGGCAGGACAGCAAGGGGGAGGATTGGGAAGACAATAGCAGGCATGCTGGGGATGCGGTGGGCTCTATGGTAGGGATAACAGGGTAATGTTTAAACTATAAAATGCAAGGTGCTGCTCAAAAAATCAGGCAAAGCCTCGCGCAAAAAAGAAAGCACATCGTAGTCATGCTCATGCAGATAAAGGCAGGTAAGCTCCGGAACCACCACAGAAAAAGACACCATTTTTCTCTCAAACATGTCTGCGGGTTTCTGCATAAACACAAAATAAAATAACAAAAAAACATTTAAACATTAGAAGCCTGTCTTACAACAGGAAAAACAACCCTTATAAGCATAAGACGGACTACGGCCATGCCGGCGTGACCGTAAAAAAACTGGTCACCGTGATTAAAAAGCACCACCGACAGCTCCTCGGTCATGTCCGGAGTCATAATGTAAGACTCGGTAAACACATCAGGTTGATTCACATCGGTCAGTGCTAAAAAGCGACCGAAATAGCCCGGGGGAATACATACCCGCAGGCGTAGAGACAACATTACAGCCCCCATAGGAGGTATAACAAAATTAATAGGAGAGAAAAACACATAAACACCTGAAAAACCCTCCTGCCTAGGCAAAATAGCACCCTCCCGCTCCAGAACAACATACAGCGCTTCCACAGCGGCAGCCATAACAGTCAGCCTTACCAGTAAAAAAGAAAACCTATTAAAAAAACACCACTCGACACGGCACCAGCTCAATCAGTCACAGTGTAAAAAAGGGCCAAGTGCAGAGCGAGTATATATAGGACTAAAAAATGACGTAACGGTTAAAGTCCACAAAAAACACCCAGAAAACCGCACGCGAACCTACGCCCAGAAACGAAAGCCAAAAAACCCACAACTTCCTCAAATCGTCACTTCCGTTTTCCCACGTTACGTCACTTCCCATTTTAAGAAAACTACAATTCCCAACACATACAAGTTACTCCGCCCTAAAACCTACGTCACCCGCCCCGTTCCCACGCCCCGCGCCACGTCACAAACTCCACCCCCTCATTATCATATTGGCTTCAATCCAAAATAAGGTATATTATTGATGATGTTAATTAATTTAAATTCGAACATGCATGGATCCATATGCGGTGTGAAATACCGCACAGATGCGTAAGGAGAAAATACCGCATCAGGCGCTCTTCCGCTTCCTCGCTCACTGACTCGCTGCGCTCGGTCGTTCGGCTGCGGCGAGCGGTATCAGCTCACTCAAAGGCGGTAATACGGTTATCCACAGAATCAGGGGATAACGCAGGAAAGAACATGTGAGCAAAAGGCCAGCAAAAGGCCAGGAACCGTAAAAAGGCCGCGTTGCTGGCGTTTTTCCATAGGCTCCGCCCCCCTGACGAGCATCACAAAAATCGACGCTCAAGTCAGAGGTGGCGAAACCCGACAGGACTATAAAGATACCAGGCGTTTCCCCCTGGAAGCTCCCTCGTGCGCTCTCCTGTTCCGACCCTGCCGCTTACCGGATACCTGTCCGCCTTTCTCCCTTCGGGAAGCGTGGCGCTTTCTCATAGCTCACGCTGTAGGTATCTCAGTTCGGTGTAGGTCGTTCGCTCCAAGCTGGGCTGTGTGCACGAACCCCCCGTTCAGCCCGACCGCTGCGCCTTATCCGGTAACTATCGTCTTGAGTCCAACCCGGTAAGACACGACTTATCGCCACTGGCAGCAGCCACTGGTAACAGGATTAGCAGAGCGAGGTATGTAGGCGGTGCTACAGAGTTCTTGAAGTGGTGGCCTAACTACGGCTACACTAGAAGGACAGTATTTGGTATCTGCGCTCTGCTGAAGCCAGTTACCTTCGGAAAAAGAGTTGGTAGCTCTTGATCCGGCAAACAAACCACCGCTGGTAGCGGTGGTTTTTTTGTTTGCAAGCAGCAGATTACGCGCAGAAAAAAAGGATCTCAAGAAGATCCTTTGATCTTTTCTACGGGGTCTGACGCTCAGTGGAACGAAAACTCACGTTAAGGGATTTTGGTCATGAGATTATCAAAAAGGATCTTCACCTAGATCCTTTTAAATTAAAAATGAAGTTTTAAATCAATCTAAAGTATATATGAGTAAACTTGGTCTGACAGTTACCAATGCTTAATCAGTGAGGCACCTATCTCAGCGATCTGTCTATTTCGTTCATCCATAGTTGCCTGACTCCCCGTCGTGTAGATAACTACGATACGGGAGGGCTTACCATCTGGCCCCAGTGCTGCAATGATACCGCGAGACCCACGCTCACCGGCTCCAGATTTATCAGCAATAAACCAGCCAGCCGGAAGGGCCGAGCGCAGAAGTGGTCCTGCAACTTTATCCGCCTCCATCCAGTCTATTAATTGTTGCCGGGAAGCTAGAGTAAGTAGTTCGCCAGTTAATAGTTTGCGCAACGTTGTTGCCATTGCTGCAGCCATGAGATTATCAAAAAGGATCTTCACCTAGATCCTTTTCACGTAGAAAGCCAGTCCGCAGAAACGGTGCTGACCCCGGATGAATGTCAGCTACTGGGCTATCTGGACAAGGGAAAACGCAAGCGCAAAGAGAAAGCAGGTAGCTTGCAGTGGGCTTACATGGCGATAGCTAGACTGGGCGGTTTTATGGACAGCAAGCGAACCGGAATTGCCAGCTGGGGCGCCCTCTGGTAAGGTTGGGAAGCCCTGCAAAGTAAACTGGATGGCTTTCTTGCCGCCAAGGATCTGATGGCGCAGGGGATCAAGCTCTGATCAAGAGACAGGATGAGGATCGTTTCGCATGATTGAACAAGATGGATTGCACGCAGGTTCTCCGGCCGCTTGGGTGGAGAGGCTATTCGGCTATGACTGGGCACAACAGACAATCGGCTGCTCTGATGCCGCCGTGTTCCGGCTGTCAGCGCAGGGGCGCCCGGTTCTTTTTGTCAAGACCGACCTGTCCGGTGCCCTGAATGAACTGCAAGACGAGGCAGCGCGGCTATCGTGGCTGGCCACGACGGGCGTTCCTTGCGCAGCTGTGCTCGACGTTGTCACTGAAGCGGGAAGGGACTGGCTGCTATTGGGCGAAGTGCCGGGGCAGGATCTCCTGTCATCTCACCTTGCTCCTGCCGAGAAAGTATCCATCATGGCTGATGCAATGCGGCGGCTGCATACGCTTGATCCGGCTACCTGCCCATTCGACCACCAAGCGAAACATCGCATCGAGCGAGCACGTACTCGGATGGAAGCCGGTCTTGTCGATCAGGATGATCTGGACGAAGAGCATCAGGGGCTCGCGCCAGCCGAACTGTTCGCCAGGCTCAAGGCGAGCATGCCCGACGGCGAGGATCTCGTCGTGACCCATGGCGATGCCTGCTTGCCGAATATCATGGTGGAAAATGGCCGCTTTTCTGGATTCATCGACTGTGGCCGGCTGGGTGTGGCCGACCGCTATCAGGACATAGCGTTGGCTACCCGTGATATTGCTGAAGAGCTTGGCGGCGAATGGGCTGACCGCTTCCTCGTGCTTTACGGTATCGCCGCTCCCGATTCGCAGCGCATCGCCTTCTATCGCCTTCTTGACGAGTTCTTCTGAATTTTGTTAAAATTTTTGTTAAATCAGCTCATTTTTTAACCAATAGGCCGAAATCGGCAAAATCCCTTATAAATCAAAAGAATAGACCGAGATAGGGTTGAGTGTTGTTCCAGTTTGGAACAAGAGTCCACTATTAAAGAACGTGGACTCCAACGTCAAAGGGCGAAAAACCGTCTATCAGGGCGATGGCCCACTACGTGAACCATCACCCTAATCAAGTTTTTTGGGGTCGAGGTGCCGTAAAGCACTAAATCGGAACCCTAAAGGGAGCCCCCGATTTAGAGCTTGACGGGGAAAGCCGGCGAACGTGGCGAGAAAGGAAGGGAAGAAAGCGAAAGGAGCGGGCGCTAGGGCGCTGGCAAGTGTAGCGGTCACGCTGCGCGTAACCACCACACCCGCCGCGCTTAATGCGCCGCTACAGGGCGCGTCCATTCGCCATTCAGGATCGAATTAATTC

Primers for sequence: 5’ CGCAAATGGGCGGTAGGCGTG; 3’ CCTCTACAAATGTGGTATGGC
